# Analysis of Gait Biomechanics in Patients After Total Hip and Knee Arthroplasty Using Low-Cost Sensors: An Observational Repeated-Measures Study

**DOI:** 10.3390/s26092731

**Published:** 2026-04-28

**Authors:** Lea Atelšek, Matic Sašek, Žiga Kozinc

**Affiliations:** 1 Faculty of Heatlh Sciences, University of Primorska, SI-6310 Izola, Sloveniamatic.sasek@fvz.upr.si (M.S.); 2 Ludwig Boltzmann Institute for Rehabilitation Research, A-1140 Vienna, Austria

**Keywords:** RunScribe sensors, total hip arthroplasty, total knee arthroplasty, gait pattern

## Abstract

Osteoarthritis is a leading cause of lower-limb arthroplasty, and although total hip arthroplasty (THA) and total knee arthroplasty (TKA) reduce pain and improve quality of life, gait impairments often persist after surgery. This study aimed to analyze gait patterns in individuals following THA and TKA using the wearable RunScribe™ sensor system and to examine its sensitivity to short-term changes during rehabilitation. Thirty-seven patients (19 THA, 18 TKA) attending a two-week inpatient rehabilitation program were assessed twice, on the first and final day of rehabilitation. Gait was measured during a 2 min circular walk test, and both global spatiotemporal variables and limb-specific loading-related variables were analyzed. A significant main effect of time was observed for walking speed (*p* = 0.001, ηp^2^ = 0.284), with improvements of approximately 10% in both groups, as well as for step cadence (*p* < 0.001, ηp^2^ = 0.429) and contact time (*p* < 0.001, ηp^2^ = 0.380). Loading-related variables also changed significantly over time, including impact acceleration (*p* = 0.004, ηp^2^ = 0.226), braking acceleration (*p* < 0.001, ηp^2^ = 0.419), and rate of force development (*p* < 0.001, ηp^2^ = 0.412). No statistically significant between-group differences were observed for global gait variables, although participants following THA showed a tendency toward better walking performance (e.g., higher cadence, *p* = 0.065). These findings suggest that early rehabilitation is associated with measurable improvements in gait after arthroplasty and support the potential of affordable wearable sensors as practical tools for objective gait assessment in clinical settings.

## 1. Introduction

Total hip or knee arthroplasty is a common surgical procedure for osteoarthritis (OA) in these joints, a degenerative disease whose prevalence is increasing with population ageing. Among the major joints of the body, the knee is most affected by OA, followed by the hip [1]. The increasing prevalence of OA is reflected in the growing number of arthroplasty procedures performed. General indications for arthroplasty include pain, functional impairment, radiographic changes and unsuccessful conservative treatment [2]. The main aim of arthroplasty is to reduce pain, improve mobility, and restore the function of the affected limb, particularly in the performance of daily activities. Many physiotherapy interventions are aimed at restoring a normal gait pattern or improving walking ability, as these have an important impact on quality of life. For this reason, gait is frequently analyzed during rehabilitation after arthroplasty, as it allows researchers to detect even subtle deviations and thereby obtain information relevant to improving patient function after arthroplasty [3]. In clinical practice, observational gait analysis is a key component in forming an initial impression of an individual’s gait pattern and serves as a basis for further assessment [4]. Quantitative gait analysis, however, enables more precise identification of potential postoperative pathologies or deviations and serves as a tool for evaluating the effectiveness of therapeutic interventions [5].

Despite pain reduction and improved quality of life following arthroplasty, gait often remains altered after surgery [6]. Although individuals are generally able to walk faster than before surgery [7,8], previous studies have reported various deficits compared with healthy individuals. In line with earlier research [9,10,11], individuals following total hip arthroplasty (THA) have been shown to walk with lower step cadence, reduced walking speed, longer stance phase duration, and prolonged double-support time compared with healthy controls [12,13]. Asymmetries also emerge in comparison with the non-operated leg, either because of improper loading before surgery or because of the operation itself. Altered gait patterns and the associated deficits may persist for as long as two years after arthroplasty [14,15].

To identify the characteristics of gait patterns after arthroplasty, gait analysis is therefore an important part of the rehabilitation process. Clinical gait assessment is commonly performed through observation and simple field-based tests, such as the Timed Up and Go test, the 10-metre walk test, or the six-minute walk test; however, these do not allow detailed gait analysis. As noted above, the literature emphasizes the importance of quantitative gait analysis, which, compared with observational gait assessment, provides a more objective and reproducible evaluation of movement patterns [16]. Quantitative gait analysis can be performed using a range of technologies, including laboratory-based optical motion capture systems, force plates for kinetic assessment, and depth sensor-based approaches, which are often considered reference methods due to their high accuracy and comprehensive data output. However, their use is typically limited by their cost, required infrastructure, and lack of portability [17]. Wearable sensors are a promising alternative, being simple, affordable, and reliable [18]. They have been shown to be valid for motion analysis, particularly in the assessment of spatiotemporal parameters [19,20]. Among these, the RunScribe™ system stands out by enabling the measurement of numerous kinematic, kinetic and spatiotemporal variables, and it has demonstrated good validity and reliability, particularly in running analysis [21]. Although the RunScribe™ system has primarily been validated for running analysis, its underlying sensor technology enables the capture of spatiotemporal and loading-related gait parameters, suggesting potential applicability in clinical populations. Its performance in slower walking conditions and in individuals using assistive devices remains less explored, warranting further investigation. In this study, we begin to fill this gap by preliminarily investigating its suitability for gait analysis and for detecting deficits in THA and total knee arthroplasty (TKA). Therefore, the aim of our study was to analyze gait in individuals following total hip and knee arthroplasty using the affordable RunScribe™ sensor system. In addition, we sought to examine the sensitivity of this wearable system in detecting short-term changes in gait parameters during rehabilitation. Particular emphasis was placed on its ability to capture both spatiotemporal and selected loading-related variables in a clinical population. By doing so, we aimed to evaluate the potential of low-cost wearable sensors as practical tools for objective gait assessment in real-world rehabilitation settings. The study hypothesized that (1) the wearable sensor system would detect measurable improvements in gait parameters following rehabilitation, and (2) no substantial differences in global gait adaptations would be observed between THA and TKA groups in the early postoperative phase.

## 2. Materials and Methods

### 2.1. Participants

The study included 37 patients aged 48 to 85 years who underwent rehabilitation treatment at Terme Krka Strunjan. The sample comprised two groups: 19 individuals following THA and 18 following TKA. Of the participants, 16 (43.2%) were male and 21 (56.8%) were female. In addition, 16 participants (43.2%) had undergone surgery on the left leg and 21 (56.8%) on the right leg. Individuals who used crutches while walking were included, whereas those using a walker or rollator were excluded. Prior to the measurements, the participants were informed about the procedures and potential risks, and provided written informed consent to participate. All procedures were performed in accordance with the approval of the National Medical Ethics Committee of the Republic of Slovenia (No. 0120-451/2025-2711-3).

### 2.2. Equipment and Outcome Measures

Measurements were conducted in the physiotherapy facilities of Talaso Strunjan, Terme Krka, and the participants attended the assessment on two occasions. The first assessment was conducted on the first day of rehabilitation; at that time, 19 participants were two months postoperatively, 13 were three months postoperatively, three were four months postoperatively, and two were one month postoperatively. The second assessment was carried out two weeks later, on the final day of rehabilitation. Prior to the walking assessments, bilateral range of motion of the knee or hip joint was measured using a goniometer in accordance with Jakovljević and Hlebš (2017) [22]. Absolute and relative leg length were also measured using a tape measure, following the protocol described by Jakovljević and Hlebš (2017) [22].

**Figure 1:**
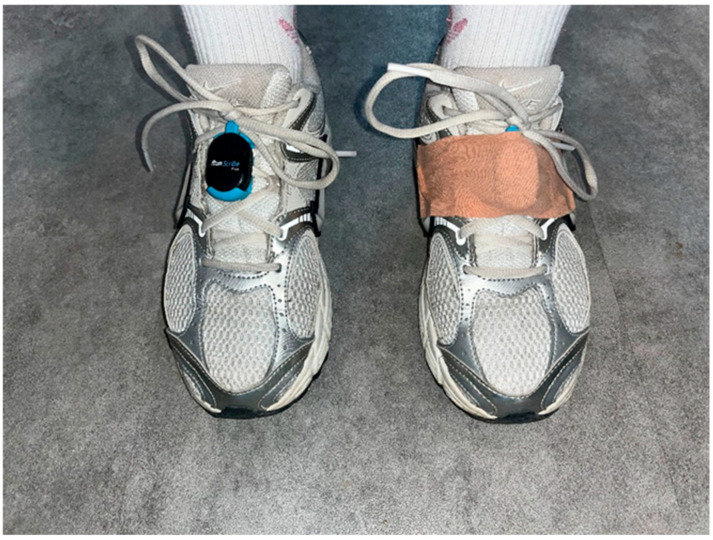
The location of the sensors and additional fixation with elastic tape.

The gait analysis was measured during the 2 min circular walk test [23], during which participants walked at a self-selected comfortable pace, using crutches if needed. Wearable RunScribe™ sensors (Scribe Labs Inc., San Francisco, CA, USA) equipped with an integrated triaxial accelerometer and gyroscope were used for data collection. Sensor placement followed the protocol described by Kozinc et al. (2024) [18]. Sensors were placed to each foot and were fixated with shoelaces. An elastic strap was added over the sensor to provide additional support (Figure 1). The additional strap fixation was used to reduce sensor movement relative to the shoe. Participants attended the assessments wearing their own footwear. RunScribe data were exported from the sensors via a smartphone application and imported into Excel files on a personal computer. In the present study, sensors were positioned on the feet to capture spatiotemporal and loading-related gait variables at the point of ground contact. This placement aligns with previous work suggesting improved sensitivity of gait-related measures when sensors are located closer to the ground [24], and represents a pragmatic approach for clinical assessment where simplicity and rapid deployment are essential. While alternative configurations (e.g., trunk-mounted or multi-sensor systems) may provide more comprehensive kinematic information, the chosen setup reflects a balance between measurement specificity and clinical feasibility. Prior to data collection, the RunScribe™ system was calibrated following the manufacturer’s recommended procedure, which includes walking/running over a known distance to adjust spatiotemporal measurements. This calibration step was performed to improve the accuracy of derived gait parameters.

The following global gait variables were analyzed: walking pace, step cadence, gait cycle length, swing and stance phase duration, and power. Additionally, the following limb-specific variables during contact were calculated: contact time, the rate of vertical force development, impact acceleration in the vertical direction, and braking acceleration in the horizontal direction.

During the two-week rehabilitation program, patients received exercises aimed at improving muscle strength and range of motion, hydrotherapy, gait training, physical agents for pain reduction, muscle strengthening and scar healing promotion, cryotherapy, and passive mobilization.

### 2.3. Statistical Analysis

Statistical analysis was performed using IBM SPSS Statistics software (version 26.0, IBM Corp., Armonk, NY, USA). The results are presented as mean values and standard deviations. Normality of data distribution was assessed using the Shapiro–Wilk test and visual inspection of histograms. For the analysis of global gait variables (walking speed, step cadence, step length and power), a two-way mixed analysis of variance (ANOVA) was used, including group (THA, TKA) as the between-subjects factor and time (first and second assessment) as the within-subjects factor. For variables measured separately for the operated and non-operated leg, a three-way mixed ANOVA was applied, with group (THA, TKA) as the between-subjects factor and time (first and second assessment) and leg (operated, non-operated) as within-subjects factors. Effect size was expressed as partial eta squared (ηp^2^). In cases of statistically significant time effects or interactions, paired-samples t-tests were additionally performed to further clarify the findings. These tests enabled a more precise interpretation of whether changes over time occurred within a specific group or leg. In addition to *p*-values, effect size for dependent samples (Cohen’s d) was also calculated, based on the standard deviation of the differences, allowing estimation of the magnitude and consistency of the response within the group. Relative changes between the two assessments (% change) were also calculated to facilitate further interpretation of the magnitude of differences. To assess the potential influence of crutch use, additional comparisons were performed between participants who used crutches and those who did not, using independent-samples t-tests with effect size estimation (Cohen’s d). Effect sizes were interpreted according to generally accepted guidelines (ηp^2^: small ≥ 0.01, medium ≥ 0.06, large ≥ 0.14; Cohen’s d: small ≥ 0.20, medium ≥ 0.50, large ≥ 0.80). The threshold for statistical significance was set at *p* < 0.05.

## 3. Results

Range of motion improved between assessments in both groups, with consistent increases observed across all measured directions. In participants following TKA, knee flexion increased and extension deficit decreased, while in the THA group, improvements were observed across flexion, extension, abduction, adduction, and rotational movements. Despite these improvements, joint mobility remained limited in a proportion of participants at the second assessment. Overall, these findings indicate a general trend toward functional recovery of joint mobility over the observed period, providing relevant context for the concurrent changes detected in gait parameters.

### 3.1. Global Gait Variables

Table 1 presents the descriptive statistics for global gait variables in both groups and both assessments, along with the results of the two-way mixed ANOVA. A significant and large main effect of time was observed for walking speed (F(1, 33) = 13.09, *p* = 0.001, ηp^2^ = 0.284), indicating an improvement between the first and second assessment. In the TKA and THA group walking pace improved significantly, by 10.6% (*p* = 0.026, d = 0.57) and 10.9% (*p* = 0.013, d = 0.67), respectively. The main effects of group and group × time interaction for walking pace were non-significant (*p* = 0.056 and 0.897, respectively). A significant and large main effect of time was found for step cadence (F(1, 33) = 24.78, *p* < 0.001, ηp^2^ = 0.429), indicating an increase between assessments in both groups. Step cadence increased by 8.6% in the TKA group (*p* = 0.010, d = 0.69) group and by 9.6% in the THA group (*p* < 0.001, d = 1.07). Neither the main effect of group (*p* = 0.065) nor the interaction (*p* = 0.676) was statistically significant. No statistically significant effects were observed for gait cycle length, including the main effects of time (*p* = 0.286), group (*p* = 0.503), and their interaction (*p* = 0.146). For power, the main effect of time approached statistical significance (F(1, 33) = 4.05, *p* = 0.052, ηp^2^ = 0.109), while neither the main effect of group (*p* = 0.380) nor the interaction (*p* = 0.145) were significant. Although the main effect of group did not reach statistical significance, a consistent non-significant tendency toward better performance was observed in the THA group compared to the TKA group across most analyzed variables.

### 3.2. Limb-Specific Gait Variables

A three-way mixed ANOVA revealed a consistent main effect of time across all limb-specific variables, indicating significant changes between the first and second assessment. In contrast, no significant main effects of group and no consistent interaction effects involving group were observed, suggesting a comparable pattern of change between participants following THA and TKA. A main effect of leg was observed in selected variables, indicating differences between the operated and non-operated limb.

**Figure 2:**
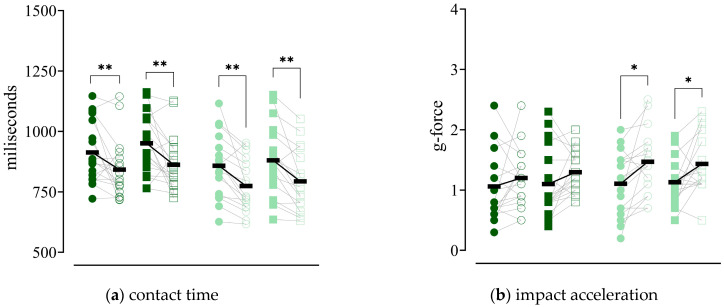

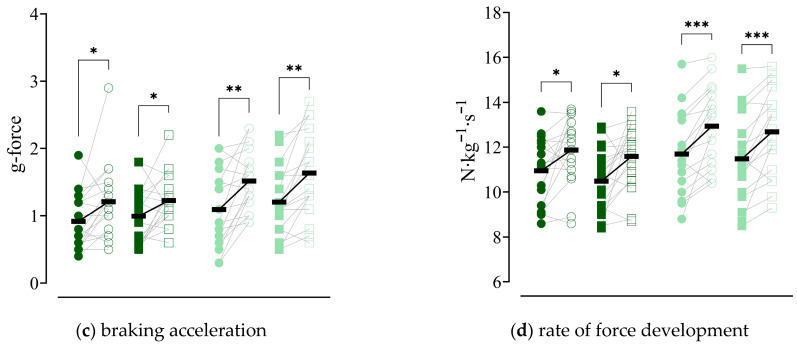
Individual differences in contact times (**a**), impact acceleration in the vertical direction (**b**), braking acceleration in the horizontal direction (**c**) and rate of vertical force development (**d**) between Assessment 1 (solid symbols) and Assessment 2 (empty symbols) for operated (circles) and non-operated limb (squares) in total knee arthroplasty (dark green) and total hip arthroplasty (light green) groups. Arithmetic mean is presented as black horizontal line. * *p* < 0.05, ** *p* < 0.01, *** *p* < 0.001.

For contact time (Figure 2a), a significant and large main effect of time was observed (F = 20.22, *p* < 0.001, ηp^2^ = 0.380), reflecting a reduction between assessments. A significant main effect of leg was also found (F = 11.57, *p* = 0.002, ηp^2^ = 0.260), with shorter contact times in the operated limb compared to the non-operated limb. Importantly, a significant time × leg interaction was identified (F = 5.10, *p* = 0.031, ηp^2^ = 0.134), indicating that changes over time differed between limbs. Descriptive results showed that contact time decreased in both limbs in both groups, with a slightly more pronounced reduction in the non-operated limb, particularly in the TKA group. Post hoc paired comparisons confirmed significant reductions in contact time for both limbs in both groups (all *p* ≤ 0.024), with moderate-to-large effect sizes (d = 0.58–0.99). No significant main effect of group (*p* = 0.098) or interactions involving group (all *p* ≥ 0.134) were observed.

For impact acceleration (Figure 2b), a significant main effect of time was observed (F = 9.65, *p* = 0.004, ηp^2^ = 0.226), reflecting increased values over time. No significant effects of group (*p* = 0.371), leg (*p* = 0.557), or interactions (all *p* ≥ 0.304) were found, suggesting a uniform pattern of change across conditions.

For braking acceleration (Figure 2c), a significant and large main effect of time was observed (F = 23.77, *p* < 0.001, ηp^2^ = 0.419), indicating increased values between assessments. In addition, a significant main effect of group was found (F = 4.47, *p* = 0.042, ηp^2^ = 0.119), with higher values in the THA group compared to the TKA group. No significant effects of leg (*p* = 0.302) or interaction effects (all *p* ≥ 0.259) were observed.

For the rate of force development (Figure 2d), a significant and large main effect of time was observed (F = 23.14, *p* < 0.001, ηp^2^ = 0.412), indicating increased values between assessments. A significant main effect of leg was also found (F = 11.18, *p* = 0.002, ηp^2^ = 0.253), with higher values in the operated limb. No significant group effect (*p* = 0.067) or interaction effects (all *p* ≥ 0.060) were observed, indicating a similar pattern of change across groups and limbs.

Overall, these results indicate that loading-related variables consistently increased over time, with minimal differences between groups and generally similar adaptations in both limbs.

## 4. Discussion

The aim of our study was to analyze gait in individuals following THA and TKA and to investigate the effects of a two-week rehabilitation program using wearable RunScribe™ sensors. The results showed statistically significant improvement in selected global gait variables between the two assessments. No statistically significant differences were observed between THA and TKA. Improvement was present in both the operated and non-operated leg, highlighting the important role of rehabilitation and physiotherapy after surgery.

The previous literature has reported similar changes in spatiotemporal gait variables in individuals following THA and TKA. In both groups, the most prominent findings are reduced walking speed, shorter step length, and prolonged stance time [25,26]. In our analysis of global spatiotemporal gait variables, no statistically significant differences were detected between participants following THA and TKA. It should, however, be noted that the results indicated a tendency towards faster walking speed (*p* = 0.056) and higher step cadence (*p* = 0.065) in individuals following THA, although these differences did not reach statistical significance. This may be explained by the fact that, in the early postoperative period, gait patterns are similar regardless of the type of surgery due to pain, reduced muscle strength, limited range of motion and use of walking aids [25]. Despite the lack of statistically significant differences in global variables between the groups, we observed a significant group effect for braking force, with patients following THA showing greater braking force than those following TKA, suggesting certain differences in gait braking biomechanics. At first glance, greater braking force could be interpreted as a sign of poorer performance; however, it is more likely related to differences in walking speed between the groups. The literature indicates that as walking speed increases, ground reaction forces also increase, including braking forces during the initial stance phase or at initial contact [27,28]. Since patients following THA in our study achieved slightly higher walking speeds, greater braking force at initial contact may be interpreted because of better walking performance. This finding may be attributed to the strategy of controlling the body’s center of mass during the early part of the stance phase, or at initial contact, with which braking is associated.

The hip joint plays an important role in pelvic stabilization and trunk position control during walking; any change, in our case the implantation of an endoprosthesis, affects the way forces are absorbed and generated during foot–ground contact. Similar findings have been described in the existing literature, as authors report changes in gait parameters after THA, including ground reaction forces [29]. Altered loading patterns between the legs may therefore reflect adaptive mechanisms that help maintain stability and ensure more efficient force transfer during the stance phase. At the same time, greater braking forces in individuals following THA may indicate somewhat better functional performance in a comparable postoperative period, since most braking at initial contact is generated by the knee extensors, which are generally more affected after TKA than after THA. Overall, the results suggest that the type of surgery, whether THA or TKA, does not have a statistically significant effect on global spatiotemporal gait variables in the early postoperative period. In our study, the analysis of variance showed no statistically significant group effect for walking speed, step cadence, step length or power. Likewise, no statistically significant interaction between group and time was observed. The only statistically significant difference between the groups was found for braking force, with mean values being higher in the THA group. Nevertheless, the THA group also showed a non-significant tendency towards better walking performance and more favorable values in selected spatiotemporal gait variables. Although gait cycle length remained similar, individuals following THA walked with higher step cadence and consequently at a faster pace. Inter-individual variability in gait adaptations may also be influenced by differences in movement strategies, particularly in the redistribution of joint moments across the hip, knee, and ankle joints, as described in the context of gait control [30]. Although not directly assessed in the present study, such strategy-dependent coordination may partly contribute to variability in loading-related parameters. Although the change in power did not reach statistical significance, the observed moderate effect size (ηp^2^ = 0.109) may indicate a meaningful trend toward improved mechanical output during gait. This could reflect enhanced propulsion capacity and more effective transfer of force during the stance phase, which are relevant for functional mobility. However, given the borderline statistical result and the indirect estimation of power by the wearable system, this finding should be interpreted with caution.

Improvement in gait pattern following TKA and THA is gradual and associated with pain reduction, healing, improved strength of key muscle groups and restoration of joint range of motion. The results of our study indicate that spatiotemporal gait variables such as walking speed, cadence, and contact time also improve over time, similarly in both THA and TKA. These findings are consistent with the existing literature and previously described recovery trends after arthroplasty, where authors report faster walking, higher cadence, and a shorter gait cycle [29,31]. These changes are likely the result of gradual pain reduction, improved muscle strength, and increased range of motion, which allow more symmetrical loading of the legs during walking [32]. The greatest effect of time and rehabilitation was observed in walking speed, which improved by approximately 10% in both patients following TKA and THA. A similar pattern was observed for step cadence, which increased significantly in both groups. At the same time, the concurrent reduction in contact time suggests a more dynamic and fluent gait pattern after the two-week rehabilitation program. In the literature, increased walking speed is often regarded as an important indicator of functional recovery, as it is closely associated with independence and the ability to perform activities of daily living [33,34].

Interestingly, gait cycle length did not change significantly over time in our study. This suggests that the improvement in walking speed occurred because of increased cadence and shorter contact time rather than longer steps. Some authors have reported similar findings [29], whereas others describe that individuals may increase walking speed either by increasing cadence or by increasing step length; thus, in certain pathologies, particularly in people with movement limitations, speed increases primarily through a higher step cadence [35,36].

Statistically significant changes across multiple spatiotemporal and loading-related variables indicate that the wearable system was sensitive to short-term alterations in gait patterns in postoperative patients. Improvements in walking speed and cadence, accompanied by reduced contact time, suggest that the sensors were able to capture meaningful changes in gait dynamics over a relatively short monitoring period. These findings highlight the capability of wearable RunScribe™ sensors to detect clinically relevant changes in both global and limb-specific gait parameters, including subtle adaptations in loading characteristics. The observed consistency of changes across variables further supports the responsiveness of the system in a rehabilitation context. From a measurement perspective, wearable sensors provide an accessible alternative to laboratory-based gait analysis, enabling objective quantification of movement patterns in real-world clinical settings. Their ease of use, low cost, and rapid deployment allow for repeated assessments without the constraints of specialized laboratory equipment. This facilitates more frequent and ecologically valid monitoring of gait, which may be particularly valuable in tracking recovery trajectories. Overall, the results support the potential of wearable sensor systems as practical tools for objective gait assessment, capable of detecting both spatiotemporal and kinetic changes in patients following lower-limb arthroplasty.

This study also has several limitations, including the small sample size, the absence of preoperative measurements and a control group, and the relatively short follow-up period, all of which limit generalizability and insight into long-term recovery. Given the short observation period, only two assessment time points, and variability in postoperative stage at baseline, stage-specific recovery patterns could not be examined. The absence of a control group limits the interpretation of the findings, as it is not possible to determine whether the observed gait parameters approach normative values or represent full recovery following arthroplasty. Future studies should include age-matched healthy controls to better contextualize postoperative gait patterns. No a priori sample size calculation was performed, as the study was designed as an exploratory clinical investigation; future studies should consider formal sample size estimation based on expected effect sizes to strengthen statistical inference. In addition, we did not consider certain potentially relevant factors that may influence gait pattern, such as pain level, muscle strength, type of surgical approach, or patients’ physical fitness, and the results may also have been affected by differences in the measurement protocol. An additional limitation is the variability in postoperative time at baseline (1–4 months), which introduces heterogeneity in recovery status across participants. As gait characteristics are known to evolve during the postoperative period, this variability may have influenced the magnitude and consistency of observed changes. Moreover, potentially relevant factors such as pain level, muscle strength, surgical approach, and other clinical characteristics were not controlled or consistently available, which may have contributed to inter-individual variability in gait patterns. A further limitation is the use of sensors designed primarily for running, which may be less sensitive in detecting very slow walking or walking with assistive devices. Although the RunScribe™ system has demonstrated acceptable validity for several spatiotemporal gait parameters when compared with established reference systems in previous studies, the accuracy of derived biomechanical variables may vary depending on the specific metric and underlying algorithms. As the system relies on proprietary data processing and indirect estimation of certain variables, the results should be interpreted with appropriate caution, particularly for loading-related parameters. In addition, test–retest reliability was not assessed in the present study, as the primary aim was to examine responsiveness to change. These factors should be considered when interpreting the findings.

## 5. Conclusions

This study investigated gait patterns following THA and TKA, as well as the effects of a two-week rehabilitation program, using affordable wearable sensors. The findings highlight the importance of early rehabilitation and suggest that wearable sensors may serve as a useful tool for the objective monitoring of gait in clinical practice. No statistically significant differences in global spatiotemporal gait variables were found between the two groups, indicating similar gait patterns in the early postoperative period. Although patients following total hip arthroplasty showed (statistically non-significant) tendency towards slightly better performance. Statistically significant improvements over time were observed in most variables: after two weeks of rehabilitation, patients walked faster, with higher cadence and shorter contact time, reflecting a more dynamic and fluent gait pattern. The study provides important insight into gait changes following total hip and knee arthroplasty and supports the usefulness of RunScribe™ wearable sensors for monitoring rehabilitation progress in clinical practice. Future studies should include larger sample sizes, age-matched control groups, and longer follow-up periods to provide a more comprehensive understanding of gait recovery trajectories following arthroplasty.

## Figures and Tables

**Table 1:** Descriptive statistics and results of two-way mixed ANOVA for global gait variables (group × time).

Variable	Group	Assessment 1	Assessment 2	Group Effect	Time Effect	Group × Time
Walking pace (min/km)	TKA	16.73 ± 3.21	14.95 ± 2.39	*p* = 0.056 ηp^2^ = 0.106	*p* = 0.001 ηp^2^ = 0.284	*p* = 0.897 ηp^2^ = 0.001
	THA	15.14 ± 3.07	13.49 ± 1.72			
Step cadence (steps/min)	TKA	86.22 ± 9.18	93.67 ± 9.98	*p* = 0.065 ηp^2^ = 0.100	*p* < 0.001 ηp^2^ = 0.429	*p* = 0.676 ηp^2^ = 0.005
	THA	92.00 ± 13.12	100.82 ± 11.90			
Gait cycle length (m)	TKA	1.52 ± 0.02	1.54 ± 0.02	*p* = 0.503 ηp^2^ = 0.014	*p* = 0.286 ηp^2^ = 0.034	*p* = 0.146 ηp^2^ = 0.063
	THA	1.54 ± 0.02	1.53 ± 0.03			
Power (W)	TKA	73.60 ± 23.25	75.50 ± 10.80	*p* = 0.380 ηp^2^ = 0.023	*p* = 0.052 ηp^2^ = 0.109	*p* = 0.145 ηp^2^ = 0.063
	THA	72.40 ± 13.32	85.50 ± 22.40			

Values are presented as mean ± standard deviation. A two-way mixed ANOVA was performed with group (TKA, THA) as the between-subjects factor and time (Assessment 1, Assessment 2) as the within-subjects factor. Effect sizes are reported as partial eta squared (ηp^2^).

## Data Availability

The data presented in this study are available on request from the corresponding author.

## Abbreviations

The following abbreviations are used in this manuscript:

OA	Osteoarthritis
THA	Total Hip Arthroplasty
TKA	Total Knee Arthroplasty
